# Untargeted metabolomics reveals sour jujube kernel benefiting the nutritional value and flavor of *Morchella esculenta*

**DOI:** 10.1515/biol-2022-0708

**Published:** 2023-08-31

**Authors:** Fenfang Wu, Zhiyuan Li, Xiaoni Chen, Xinlei Si, Shan Lin

**Affiliations:** Department of Central Laboratory, Shenzhen Hospital, Beijing University of Chinese Medicine, Shenzhen, Guangdong, China; Department of Acupuncture, Shenzhen Hospital, Beijing University of Chinese Medicine, Shenzhen, Guangdong, China

**Keywords:** *Morchella esculenta*, metabolomics, nutritional value, biological activity

## Abstract

Nucleosides, organic acids, and amino acids separated from *Morchella esculenta* are well known for their nutritional value and flavor. However, how to increase their content in a better way has been a challenge. In this study, the effect of adding jujube kernel on the active components of *M. esculenta* was investigated by untargeted metabolomics using UPLC-MS/MS. A total of 1,243 metabolites were identified, of which 262 metabolites (21.078%) were organic acids and derivatives, 245 metabolites (19.71%) were lipids and lipid-like molecules, and 26 metabolites (2.092%) were nucleosides, nucleotides, and analogues. Subsequently, differential metabolites between groups were screened by the orthogonal partial least squares-discriminant analysis model, which showed that 256 metabolites were identified as significantly different for the positive ion model and 149 for the negative ion model. Moreover, significant differential metabolites (VIP > 1, *P* < 0.05) in annotation of kyoto encyclopedia of genes and genomes pathway were investigated, which showed that ABC transporters were the most commonly observed transporters, followed by pyrimidine metabolism and purine metabolism. The results indicated that the main components of jujube kernel might be conducive to the accumulation of nucleoside organic acids and amino acid metabolites in *M. esculenta*. These results provide important information for the understanding of more suitable way for cultivation of *M. esculenta*.

## Graphical abstract

**Figure:**
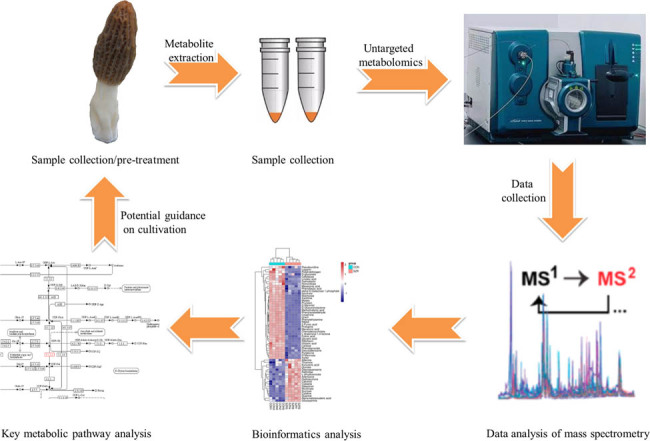

## 1 Introduction

*Morchella esculenta* (also known as “Yellow morel”) is a widely distributed fungus all over the world, especially in France, Germany, USA, India, and China, which is generally found at low altitude plains to high altitudes of about 3,200 m, mostly growing in the humus layer of broad-leaved forests or coniferous and mixed broad-leaved forests [1]. *M. esculenta* is generally divided into 28 species and belongs to the species of *Morchella*, genus of *Morchellaceae*, family of *Oncobacteria*, subdivision of *Ascomycetes*, which has been valued for its high nutritional, medicinal, and physiological functions [2]. Recently, the biological activity of *M. esculenta* derived compounds has attracted attention, which has many functional properties including antibacterial [3], anticancer [4,5], and anti-inflammatory [6]. Moreover, current research demonstrated a wide range of biological functions including antioxidant activities [7,8], immunomodulatory activities [9], hepatoprotective effects [10], and anti-melanogenesis effects [11]. Due to health benefits and flavor, morels are not only provided as dishes but also used in skin care products. Recently, it has been cultivated and matured in our country and can be sold in large quantities [12].

Suanzao kernel is the dried seed of *Jujube genus* in the rhamnus family [13]. It has the functions of calming the heart, nourishing the liver, collecting sweat, and generating fluid [14]. Generally, more than 100 kinds of chemical components have been isolated and identified from sour jujube kernel, among which saponins are the main components [15]. Jujube saponins can be divided into tetracyclic triterpenes and pentacyclic triterpenes according to the structure of aglycones. At present, over 30 saponins have been identified from jujube kernel [16]. Current pharmacological studies showed that saponins are the main active components of jujube kernel [17,18]. Furthermore, the fermentation medium of *Kombucha* SCOBY with jujube kernel enhanced its sedative and hypnotic effect, which provided a new approach to process Chinese medicine with better functional effects and acceptable flavor [19]. Moreover, the addition of jujube kernel to the liquid fermentation medium of *Schizophyllum commune* Fr showed that the effective biomass, as well as the crude polysaccharide and total saponin content were significantly increased. Therefore, adding jujube kernel and saponins in jujube kernel into culture medium of *M. esculenta* may have positive effects on the metabolites and nutrient composition.

On the basis of genome, transcriptome, proteome, and other omics, metabolome is considered as a new “histology” [20]. At present, metabolomics has been widely used in many fields. Metabolomics was used to compare the metabolic amount between the experimental group and the control group to find out the differential metabolites, which can help in the screening of biomarkers, or to study the biological processes involved in the differential metabolites, to reveal the mechanisms of their involvement in life activities, and to completely study the regulatory pathways [21]. According to the detection mode, the metabolome can be divided into two categories: untargeted and targeted [22]. Non-targeted detection technologies are mostly based on high-resolution mass spectrometry, which can realize unbiased, large-scale and systematic detection of various metabolites in samples, providing an “aerial” view to reflect the maximum perturbation of metabolic levels in organisms, and therefore suitable for pre-project basic research [23]. It is suitable for metabolite detection of specific interest and validation of non-target metabolomes.

In order to find out a better way of cultivation, this article studied the various cultivation ways of *Morchella*. Subsequently, high-resolution non-targeted metabolomics technology was applied to detect metabolites in samples using ultra-high performance liquid chromatography-tandem time-of-flight mass spectrometry (UHPLC-Q-TOF MS), and the differential metabolite analysis may provide important information for the understanding of more suitable way to cultivate *M. esculenta*.

## 2 Materials and methods

### 2.1 Reagents and samples

Fresh morel mushrooms from two different cultivation methods were collected during their fruiting period. This matrix was first prepared by wheat (40%), mixed wood chips (30%), rice husk (20%), and humus (10%). Then, an extra lime (1%), gypsum (2%), potassium dihydrogen phosphate (0.1%), and sour jujube kernel (5%) were added. In addition, the matrix of control group has the same components except that no sour jujube kernel is added. Formic acid and ammonium acetate were commercially available from Sigma-Aldrich (Madrid, Spain). Methanol and acetonitrile were obtained from ANPEL (Shanghai, China). A Milli-Q water purification unit (Millipore, Millipore, MA, USA) was used throughout the study. The chemical reagents and solvents used in this product have been subjected to high performance liquid chromatography or analysis.

### 2.2 Sample preparations

Production conditions of morel mushrooms are described as follows: soil moisture of 60–70%, temperature of 10–20°C, sunlight transmission of 30%, soil pH requirements between 6.5 and 7.5, and the need for sufficient oxygen. After morel mushrooms were harvested, they were dried and crushed into powder form for untargeted metabolomics analysis. All frozen samples were ground to a fine powder and passed through a 425 µm sieve. Fine powder of 1.0 g was determined by UPLC-Q-TOF-MS and extracted by ultrasonic wave for 30 min. Next, the extract was centrifuged at 12,000 rpm for 20 min at 4°C. Next, the supernatant of each tube (100 µL) was filtered through a 0.22 µm micro-sieve and transferred to an LC vial. The content of the sample was determined by UPLC-Q-TOF-MS method, and the sample was stored at 4°C. In addition, all samples were equally divided into several parts, fully stirred by vortex method, and then measured by liquid chromatography to obtain quality control samples.

The sample was slowly dissolved at 4°C, the supernatant was dried *in vacuo*, and 100 μL of acetonitrile aqueous solution (acetonitrile:water = 1:1, volume ratio) was added for mass spectrometry. After centrifuging the supernatant at 14,000*g* for 15 min at 4°C, the supernatant was harvested for analysis.

### 2.3 Data acquisition of UPLC-Q-TOF-MS

The sample with hydrophilic interaction liquid chromatography (HILIC) was separated by Agilent 1290 InfinitLC (UHPLC) system at a column temperature of 25°C. The flow rate was set at 0.5 mL/min, and the injection volume was set to 2 μL, where *A* was water + ammonium acetate + ammonia water 25 mmol, *B* was acetonitrile, and gradient elution was carried out according to the following steps: 0–0.5 min, 95% *B*. Within 0.5–7 min, the *B* value changes linearly from 95 to 65%; within 7–8 min, the *B* value changes linearly from 65 to 40%; within 8–9 min, the *B* value remains at 40%; from 9 to 9.1 min, the *B* value changes linearly from 40 to 95%; from 9.1 to 12 min, the *B* value remains at 95%. During the measurement, the sample was placed at 4°C. During testing, the sample was placed in the autosampler. In order to eliminate the fluctuation of the measurement signal, the sample was analyzed in irregular continuous. On this basis, the QC samples were added to the sample library, and the experimental results were monitored and evaluated in real time.

Primary and secondary spectra were obtained using an AB TripleTOF6600 mass spectrometer. After HILIC chromatography, the ESI source conditions are Gas1 (Gas1) = 60, Gas2 (Gas2) = 60, CUR (CUR) = 30, source temperature 600°C, and IonSapary Voltage Floating ±5,500 V (positive, negative); the *m*/*z* scan (*m*/*z*) range of TOF MS is set at 60–1,000 Da, the *m*/*z* scan (*m*/*z*) range of product ions is set at 25–1,000 Da, the cumulative scan time of TOF MS is set at 0.20 s/s/s, and the cumulative scanning (*m*/*z*) time of product ions is set at 0.05 s/s. This project intends to adopt a highly sensitive and high-precision information association acquisition method, on this basis, to study the declustering potential: ±60 V (both positive and negative), collision energy (35 ± 15 eV), excluding isotopes below 4 Da, observing ten candidates per cycle.

### 2.4 Data processing and multivariate analysis

The samples in Wiff format were converted into mzXML files by ProteoWizard, and then XCMS software was used to correct the peak position of the sample, and the peak position correction and peak position extraction were performed on the sample. This project will identify the structure of the metabolites obtained by XCMS and preprocess them, then evaluate the quality of the test results, and conduct statistical analysis on the results. The specific contents of data analysis include univariate statistical analysis, multidimensional statistical analysis, screening of differential metabolites, correlation analysis of differential metabolites, and kyoto encyclopedia of genes and genomes (KEGG) path analysis (http://www.genome.jp/kegg/pathway.html).

## 3 Results

### 3.1 Quality evaluation of experimental data

The stability of the instrument, the repeatability of the experiment, and the reliability of the experiment were comprehensively evaluated. The test system designed in this article has satisfied the performance, and the test results are stable and reliable. The metabolite profile obtained from the experiment can reflect the biological characteristics of the sample itself.

The total ion current curve of the QC samples was compared with the overlaid spectral curves shown in Figure S1a and b. Through the analysis of the two methods, it is concluded that the response intensities and retention times of the peaks basically overlapped, indicating that the variation caused by the instrumental error was small during the whole experiment.

**Figure 1:**
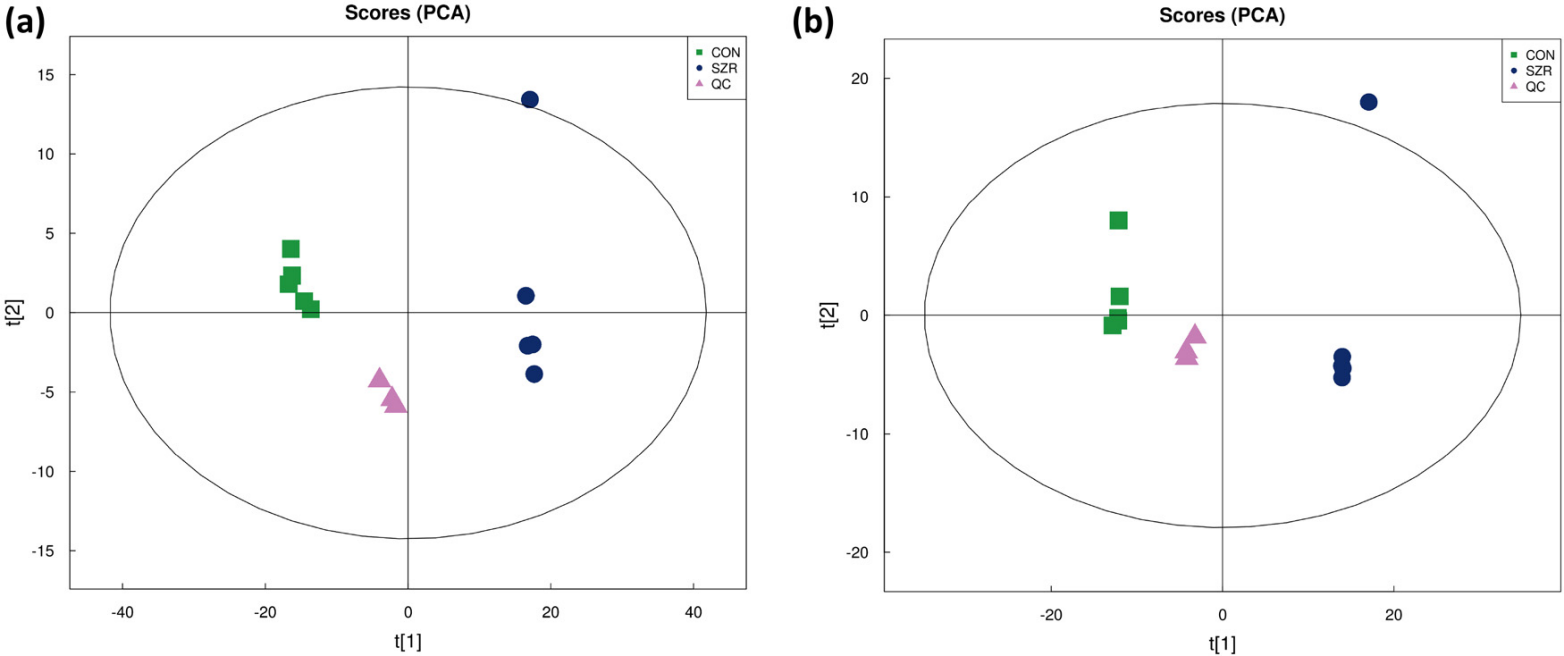
PCA analysis of QC samples: (a) PCA analysis of the overall sample in positive ion mode and (b) PCA analysis of the overall sample of negative ion patterns.

Population sample principal component analysis (PCA) was performed on the peaks extracted from the total test sample and the QC sample, as shown in Figure 1a and b. The results indicated that the proton exchange membrane showed obvious cluster structure in the positive and negative ion models, which suggested that the method had good reproducibility.

### 3.2 Identification of metabolites

**Figure 2:**
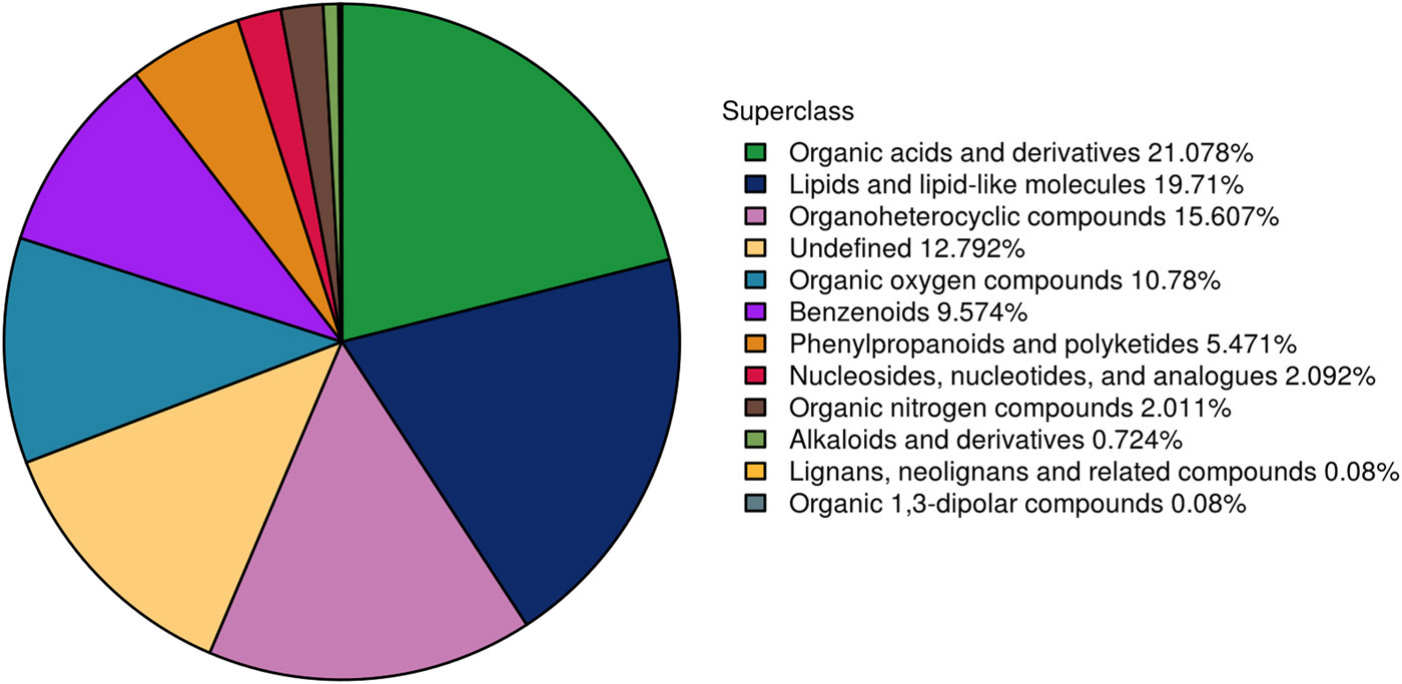
Number share of identified metabolites in each chemical classification.

In this study, the structures of match metabolites were determined in biological samples to retention times. Furthermore, the number of identified metabolites was calculated, and it was found that there were 1,243 metabolites identified by the combination of positive and negative ions, as shown in Table 1. In addition, the metabolites were chemically classified. The chemical taxonomic analysis of all metabolites (including metabolites separated by positive and negative ions) was carried out, and the distribution of various types of metabolites is shown in Figure 2.

**Table 1:** Statistics of identification of metabolites by positive and negative ion patterns

Detection mode	Number of metabolites identified
Positive ion mode (Pos)	886
Negative ion mode (Neg)	423

### 3.3 Identification of the differential metabolites

**Figure 3:**
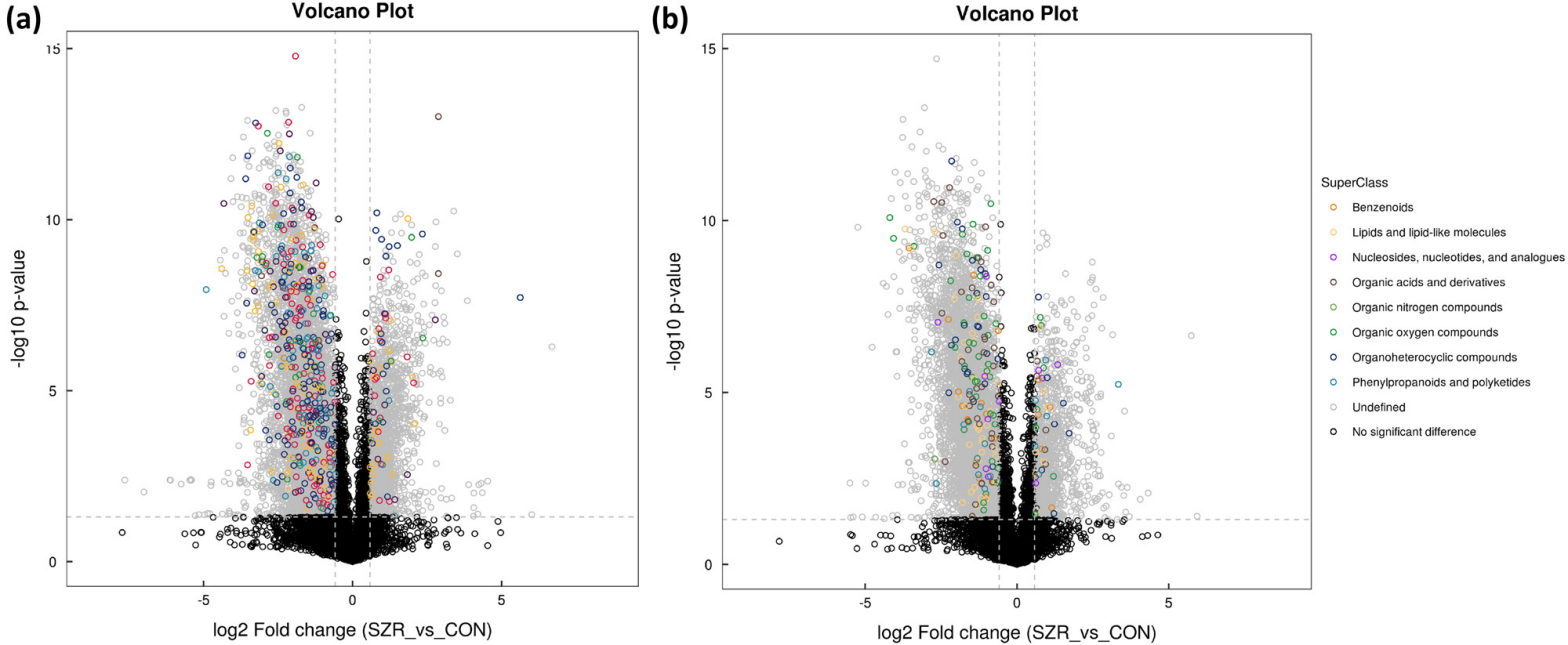
Volcano map of differential metabolite taxonomic attribution: (a) positive ion mode volcano map and (b) negative ion mode volcano map.

In this study, we analyzed the differences between the two samples by multiplier change analysis (FC analysis) and *t*-test/non-parametric test. On this basis, the differences of different types of metabolites, including unknown metabolites, in positive and negative ion modes were compared. As shown in Figure S2, the differential metabolites with FC > 1.5 or FC < 0.67, *P* value <0.05 were visualized by a volcano plot. In order to visualize the classification attribution of the differential metabolites, different colors were used to make the distinction, and the results are shown in Figure 3. By studying the metabolic pathways involved in differential metabolites and retrospectively identifying regulatory enzymes and genes, it can help to reveal the mechanisms of life activities that they are involved in.

### 3.4 Multidimensional statistical analysis

In this study, we used PCA to understand the overall distribution trend of the data and the differences between the various groups of the data. Furthermore, PCA analysis was performed for each group, and the PCA score plot of the sample vs group is shown in Figure S3. After seven cross-checks, the obtained PCA results are consistent with Table 2. Therefore, the PCA of metabolites can better reflect the differences within the group and between the groups.

**Table 2:** PCA model parameters

Ionic mode	Sample group	*A*	*R* ^2^ *X* (cum)
Positive ion mode (Pos)	SZR_vs_CON	2	0.764
Negative ion mode (Neg)	SZR_vs_CON	2	0.703

Note: *A* represents the main component score and *R* ^2^ *X* represents the model explanation rate of the *X* variable.

Orthogonal partial least squares-discriminant analysis (OPLS-DA) can enhance the resolution and accuracy of the model. In Figure S4, the OPLS-DA model scores for an exemplary control group are shown, and indicate that the OPLS-DA model is able to distinguish between the two sample groups.

**Figure 4:**
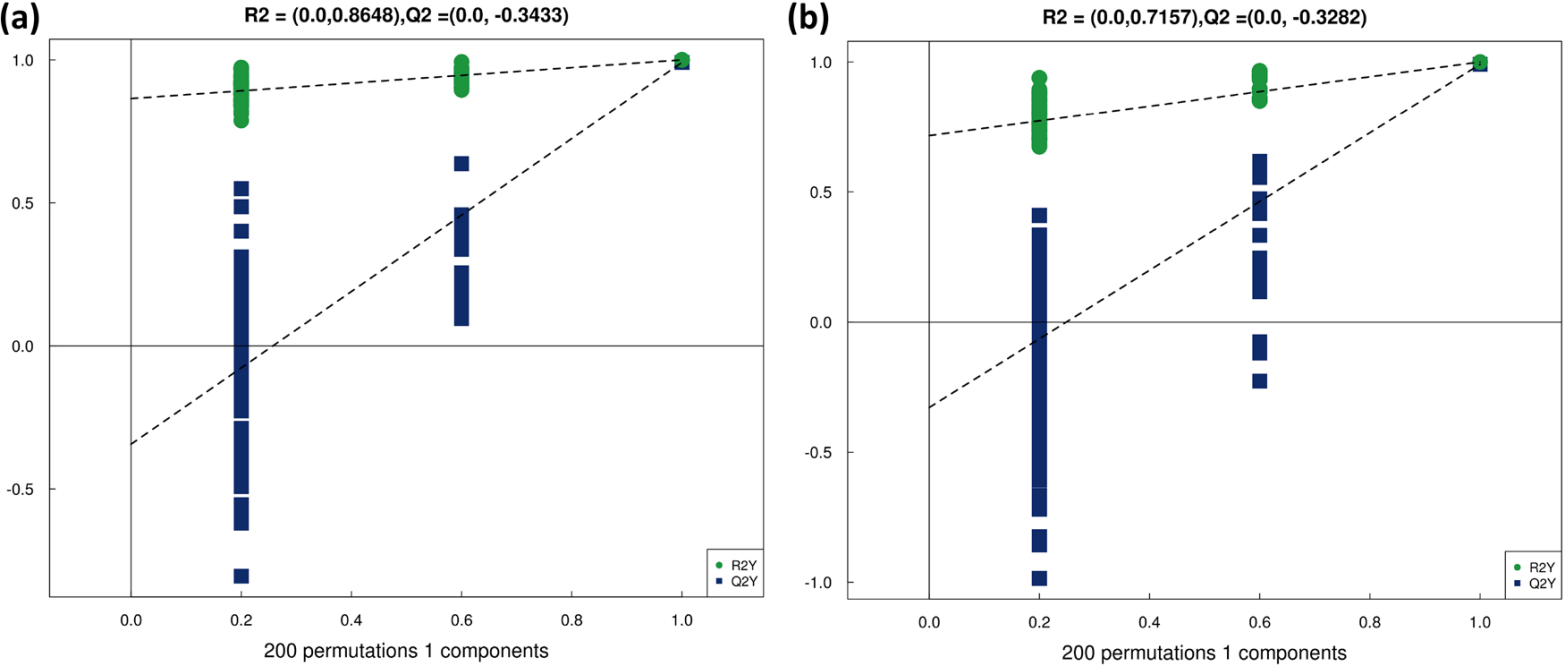
OPLS-DA displacement test results: (a) positive ion mode OPLS-DA displacement test and (b) negative ion mode OPLS-DA displacement test.

The model evaluation parameters (*R* ^2^ *Y*, *Q* ^2^) obtained by seven cross-tests are shown in Table 3. The results suggested that the model has good stability and reliability with *Q* ^2^ > 0.5. The substitution test plot for the OPLS-DA model is shown in Figure 4, which indicates that the *R* ^2^ and *Q* ^2^ of the stochastic model gradually decrease as the substitution retention rate decreases, implying that there is no overfitting in the original model and that the model is robust.

**Table 3:** Evaluation index of OPLS-DA

Ionic mode	Sample group	*A*	*R* ^2^ *X* (cum)	*R* ^2^ *Y* (cum)	*Q* ^2^ (cum)
Positive ion mode (Pos)	SZR_vs_CON	2	0.739	1	0.993
Negative ion mode (Neg)	SZR_vs_CON	2	0.7	1	0.998

Note: *R* ^2^ *Y* represents the model explanation rate of a *Y* variable and *Q* ^2^ represents the prediction of the model function.

### 3.5 Screening for differential metabolites

On this basis, we applied the OPLS-DA model to calculate the importance of each metabolite, evaluate the taxonomic differences of each metabolite in different categories, and discover the different lipid molecules with biological significances. This study was based on the two indicators of OPLS-DA VIP > 1 and *P* < 0.05 for screening metabolites with significant differences, and metabolites with VIP > 1 played an important role in the interpretation of the model. As can be seen from Figure S5, the change in the number of metabolic differences that are significantly different for ploidy recognition is represented in the bar graph.

### 3.6 Bioinformatic analysis of differential metabolites

The screened out metabolites with significant differences are subjected to subsequent original signal analysis, including correlation analysis, path analysis, and cluster analysis.

Each sample group is classified by a hierarchical method, and a cluster tree that can reflect the similarity between sample groups is obtained, and a result as shown in Figure S6 is obtained. In the same cluster, the similarity between samples is high. Obviously different metabolites (VIP > 1, *P*-value <0.05) are shown in Figure S7. Clustered metabolites, which exhibit similar expression profiles at the gene level, may have similar effects, and may also have the same metabolic pathways.

### 3.7 Correlation analysis

**Figure 5:**
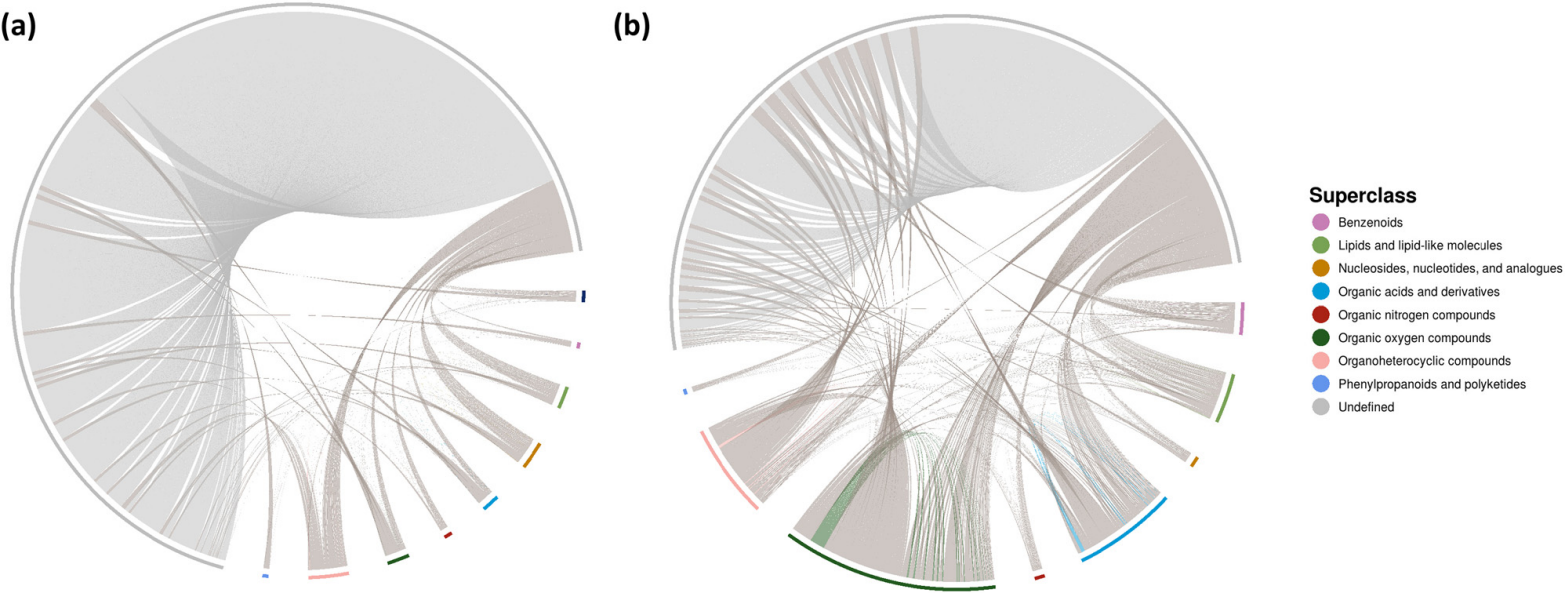
Chord diagram between metabolites: (a) positive ion mode chord diagram and (b) negative ion mode chord diagram.

According to the correlation analysis method, the correlation analysis was carried out on the metabolites with significant differences, and the results of the correlation analysis were displayed as a correlation heat map, which is shown in Figure S8. In order to make the mutual regulation among various metabolites clear, this article transforms the correlation matrix into the chord curve and network curve as shown in Figure 5 and Figure S9. The chord diagram as well as the network diagram both show that the molecular pairing of metabolites has a certain correlation, namely: *r* > 0.8, *P* < 0.05 [24], which is an indicator that can be adjusted appropriately. Among them, the chord diagram better reflects the correlation between metabolites, while diagram of the network better reflects the correlation among metabolites [25], and they have their own strengths. Therefore, correlation analysis will reveal the degree of similarity between metabolites (VIP > 1, *P* < 0.05), and provide basis for in-depth comprehending the regulatory mechanism of metabolites when physiological states change.

### 3.8 KEGG pathway annotation and analysis

In this study, the differential metabolites were marked (VIP > 1, *P* < 0.05) using the KEGG pathway map function in the KEGG pathway. Furthermore, more than five KEGG metabolic pathways were screened out, different metabolites were marked on the KEGG metabolic pathways, and the change curves of different metabolites on the KEGG metabolic pathways were drawn as a heat map, and is shown in Figure S10.

**Figure 6:**
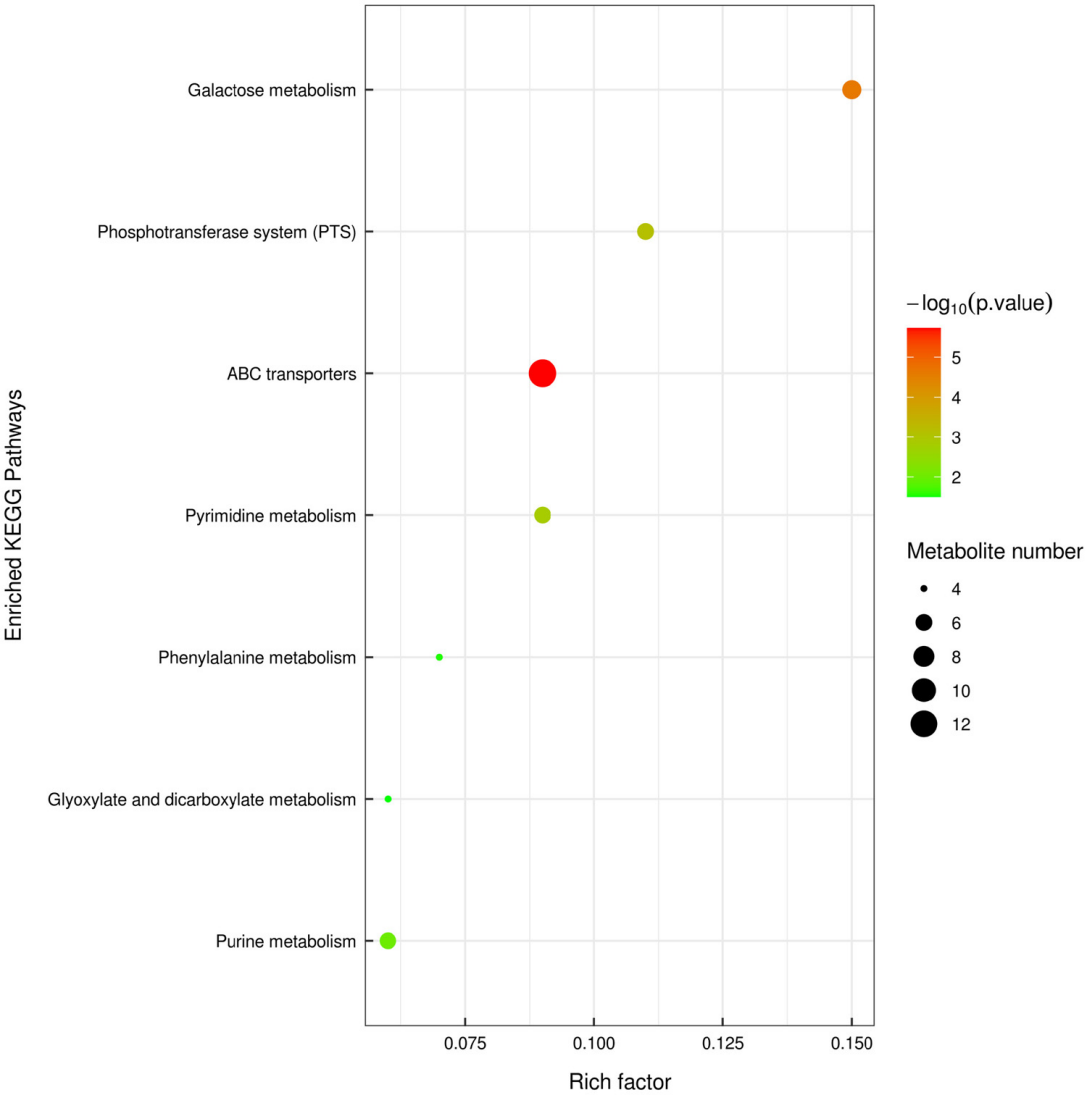
KEGG enrichment pathway diagram (bubble diagram).

In addition, the results of the metabolic pathway enrichment analysis are presented as bubble and bar graphs, and the results are shown in Figure 6. Therefore, KEGG pathway adopts Fisher’s precise test method, takes KEGG pathway and its related metabolic pathways as a whole, and calculates the significance of metabolite accumulation in each pathway, so as to find out the metabolism that is significantly affected by substances and signaling pathways.

## 4 Discussion

As a traditional fungus for both medicine and food, *M. esculenta* is widely distributed in South Korea, China, Japan, Europe, and other countries [1]. *M. esculenta* is rich in sugars, proteins, vitamins, minerals, and other ingredients. In addition, *M. esculenta* is rich in all kinds of essential amino acids, of which glutamic acid is the most, followed by aspartate and leucine [26]. Recently, *M. esculenta* is deeply favored by consumers because of its unique flavor, rich nutrition, and high medicinal value. Different medium components have different effects on the nutritional composition of *M. esculenta* [27]. In order to understand the effect of adding sour jujube kernel to the medium on the metabolism of *M. esculenta*, we developed a non-targeted metabolomics-based approach. Studies have found that adding sour jujube kernel during the cultivation process can increase the contents of organic acids, lipids, and nucleosides in *M. esculenta* thus improving the nutritional value and taste of *M. esculenta*.

Recently, UPLC-Q-TOF/MS-based method combined with multivariate statistical analysis was applied to distinguish small molecule metabolites with molecular weights <1,500 Da in a variety of organisms [28,29]. Typical food quality substances such as nucleosides, organic acids, and amino acids in *M. esculenta* can be effectively screened by non-targeted metabolomics method [30]. In this study, the metabolic profile changes of the samples were separately analyzed by metabolomics method based on UHPLC-Q-TOF MS. Quality control tests indicated that the instrument performed well and the test results were stable and reliable. The metabolite profiles obtained from the experiments could reflect the biological properties of the samples themselves.

The aroma of *M. esculenta* is mainly due to its high content of small molecules such as soluble sugars, polyols, amino acids, organic acids, etc. [31]. In *M. esculenta*, amino acids, such as alanine and glutamic acid, are important taste activators [32], which have floral aroma properties, and serine and tyrosine have wine-like properties. In general, the amino acid composition, content, and metabolites in protein have a great influence on the quality of protein. Meanwhile, organic acid is still an important substance in organisms, which plays a very important role in keeping the quality as well as nutritional value of food [31]. Furthermore, many kinds of organic acids, such as succinic acid, protect *M. esculenta* from various diseases because of their antioxidant capacity, and it also has positive effects on preservation [33]. In consequence, the results showed that amino acids as well as organic acids were the main factors in *M. esculenta* affecting its quality and flavor. In this study, both organic acids and amino acid differential metabolites showed a significant up-regulation trend in *M. esculenta*, whose medium was supplemented with sour jujube kernel, resulting in a typical *M. esculenta* flavor. Interestingly, the organic acid is dominated by a ternary or Krebs cycle, and the nutrients provided by sour jujube kernel may increase the metabolic rate and thus reduce the organic acid content.

Pyrimidine metabolism is an important form of nucleotide metabolism, which plays an important role in the bioenergetic processes and transport forms of carbohydrates such as sucrose, cellulose, cell wall matrix polysaccharides, and sugar synthesis [34]. In this study, we have systematically annotated several compounds involved in the pyrimidine metabolic pathway. Among them, the content of pyrimidine by the addition of sour jujube kernel was significantly up-regulated compared to the control group. In addition, a close link exists between organic nitrogen sources and amino acid metabolism [35]. Organic nitrogen sources may need to be supplied from external nutrient bags during the growth of hexagonal grass [36]. In this study, we conducted a systematic annotation analysis of metabolites with significantly different growth, and found significant metabolic differences involved in amino acid metabolism associated with the nitrogen source, which may be related to the added date palm enriched nitrogen source and delicately balanced during asexual growth of *M. esculenta*.

## 5 Conclusion

Regulatory differences between cultures, differential metabolites, including lipids, organic acids, and amino acids, have been shown to be viable biomarkers for differentiating regions and understanding metabolic profiles. Nevertheless, the results of this study are limited in their strength. Additional studies under controlled conditions are necessary to analyze the effects of environmental factors, intrinsic factors (including phylogeny and life cycle), and physicochemical changes in *M. esculenta* during culture/processing, particularly with regard to adenosine, organic acids, amino acids, lipids, and their detailed composition. The impact on potential applications is then assessed. Furthermore, this metabolomic analysis approach provided clear trends among different sources, which demonstrated the validity of the method. Further studies, such as absolute quantitative methods and GC/MS-based platform studies, are needed to gain insight into the effects of provenance on the nutritional value and flavor of *M. esculenta*.

## Supplementary Material

Supplementary Figure
