# Supplementary material for: Untargeted metabolomics reveals sour jujube kernel benefiting the nutritional value and flavor of Morchella esculenta
Source: Open Life Sci. 2023 Aug 31;18(1):20220708. doi: 10.1515/biol-2022-0708 (PMC10476485; doi:10.1515/biol-2022-0708)
Supplement: Supplementary Figure [file biol-2022-0708-sm.pdf]

Supplementary

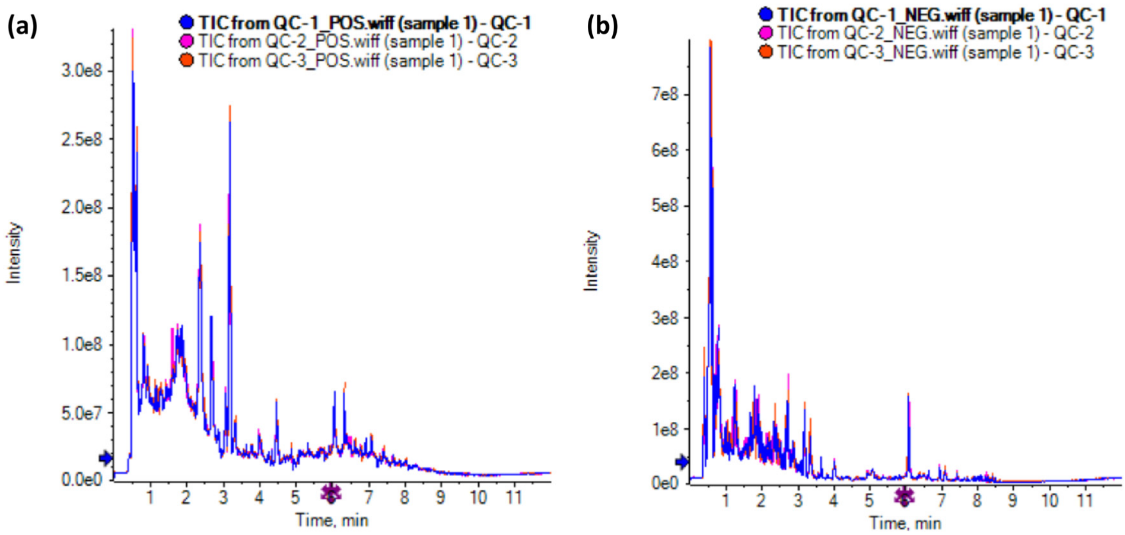

Figure S1: Total ion flow diagram of QC samples. (a) Positive ion mode QC sample total ion flow map overlapping spectra. (b) Negative ion mode QC sample total ion flow map overlay spectra. Note: The horizontal coordinates of the graph indicate the retention time of each peak and the vertical coordinates indicate the intensity values of the peaks.

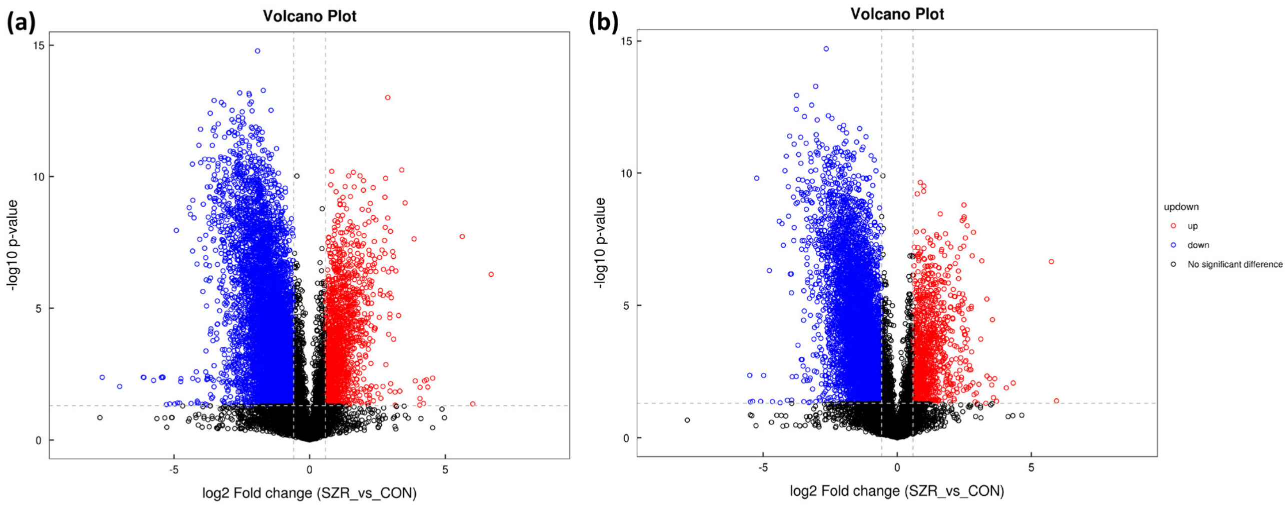

Figure S2: Volcanogram of all metabolites detected in positive and negative ion mode. (a) Positive ion mode volcano plot. (b) Negative ion mode volcano map. Note: The horizontal coordinates are the log2 values of Fold Change and the vertical coordinates are the log10 values of significant P value. Significantly different metabolites: metabolites meeting FC > 1.5 and *P* value < 0.05 are shown in red, metabolites meeting FC < 0.67 and *P* value < 0.05 are shown in blue. Non-significantly different metabolites are indicated in black.

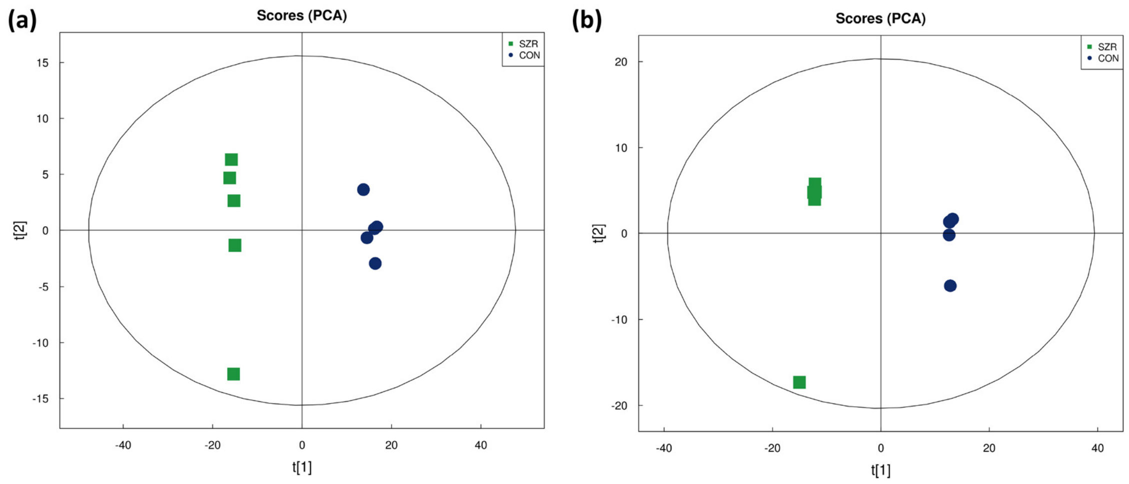

Figure S3: PCA Score Chart. (a) Positive ion mode PCA score graph. (b) Negative ion mode PCA score chart. Note: In the figure, t[1] represents principal component 1, t[2] represents principal component 2, and the ellipse represents the 95% confidence interval. The dots of the same color indicate the individual biological replicates within the group, and the distribution status of the dots reflects the degree of variation between and within groups.

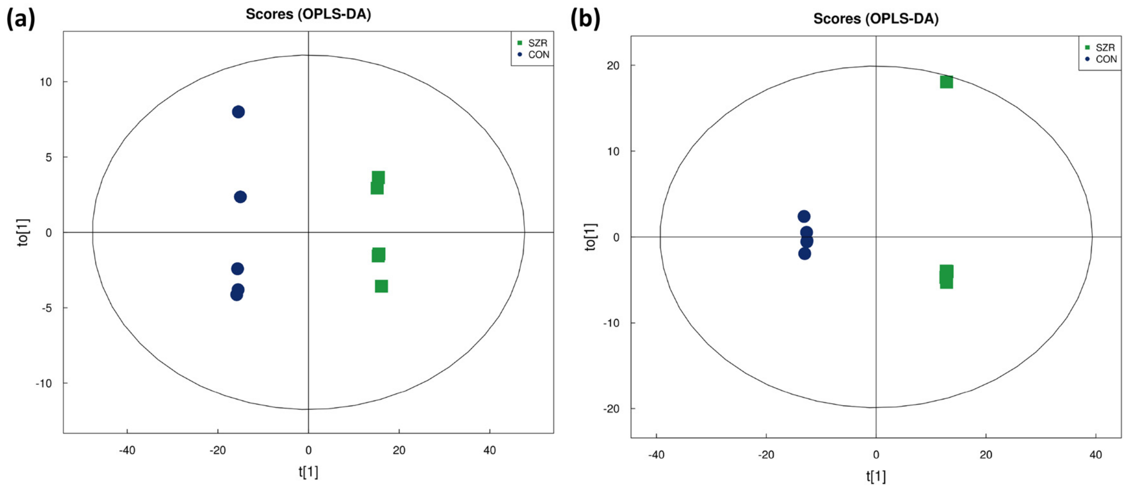

Figure S4: OPLS-DA Score Chart. (a) Positive ion mode OPLS-DA score chart. (b) Negative Ion Mode OPLS-DA Score Chart. Note: In the figure, t[1] represents principal component 1, to [1] represents principal component 2, and the ellipse represents the 95% confidence interval. The dots of the same color indicate the individual biological replicates within the group, and the distribution status of the dots reflects the degree of variation between and within groups.

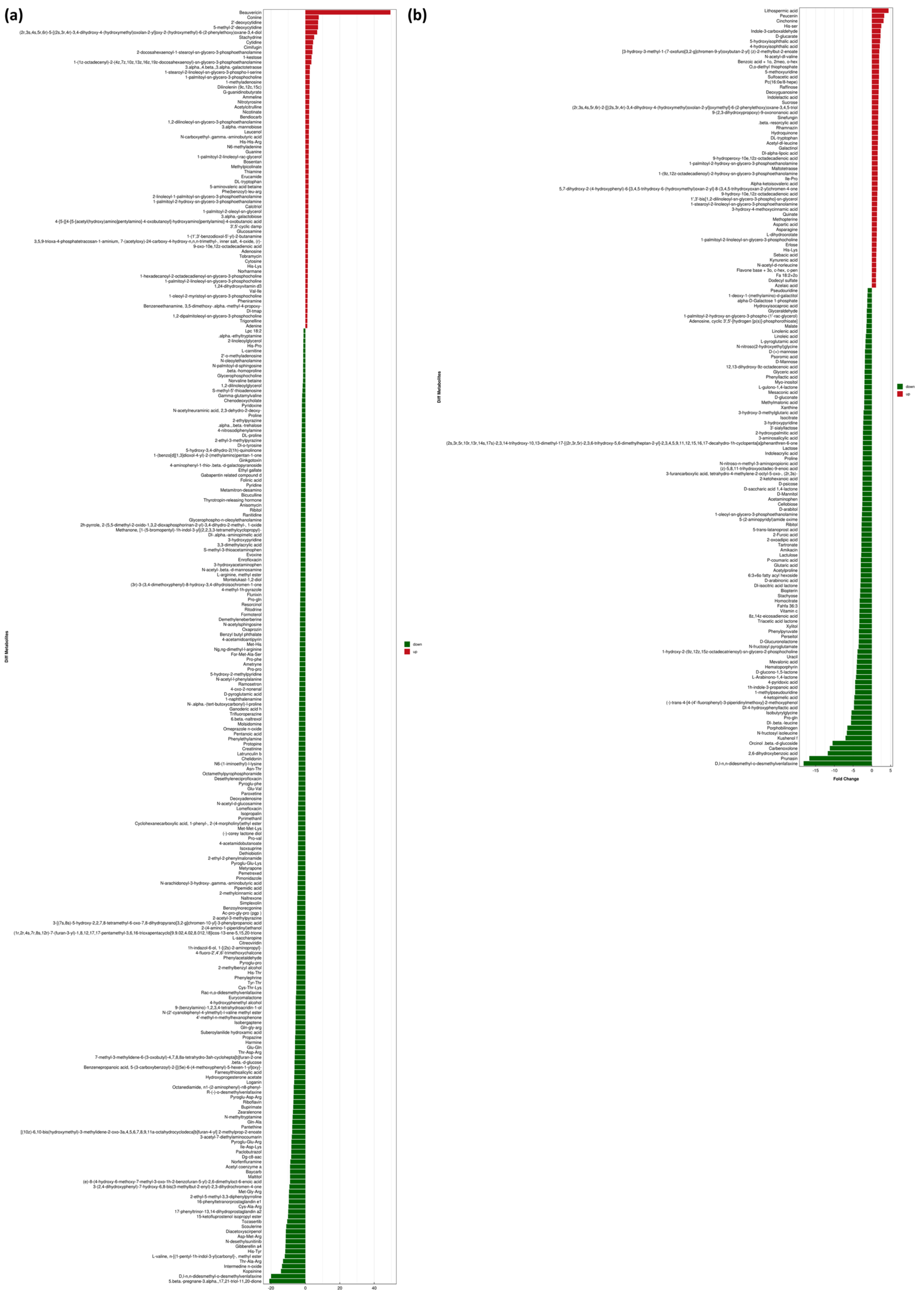

Figure S5: Significantly different metabolite expression differential ploidy analysis. (a) Positive ion pattern significant difference metabolite expression differential multiplicity analysis. (b) Analysis of differential multiplicity of metabolite expression for significant differences in negative ion patterns.

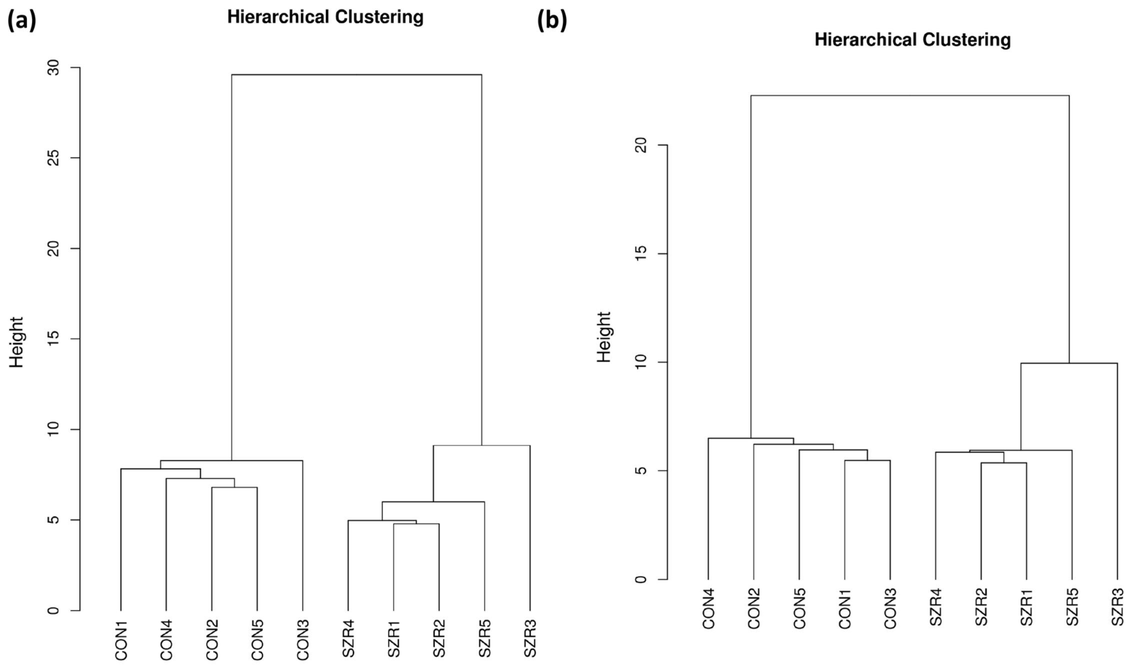

Figure S6: Sample Hierarchical Clustering Tree. (a) Positive ion pattern sample hierarchical clustering tree. (b) Hierarchical clustering tree of negative ion pattern samples.

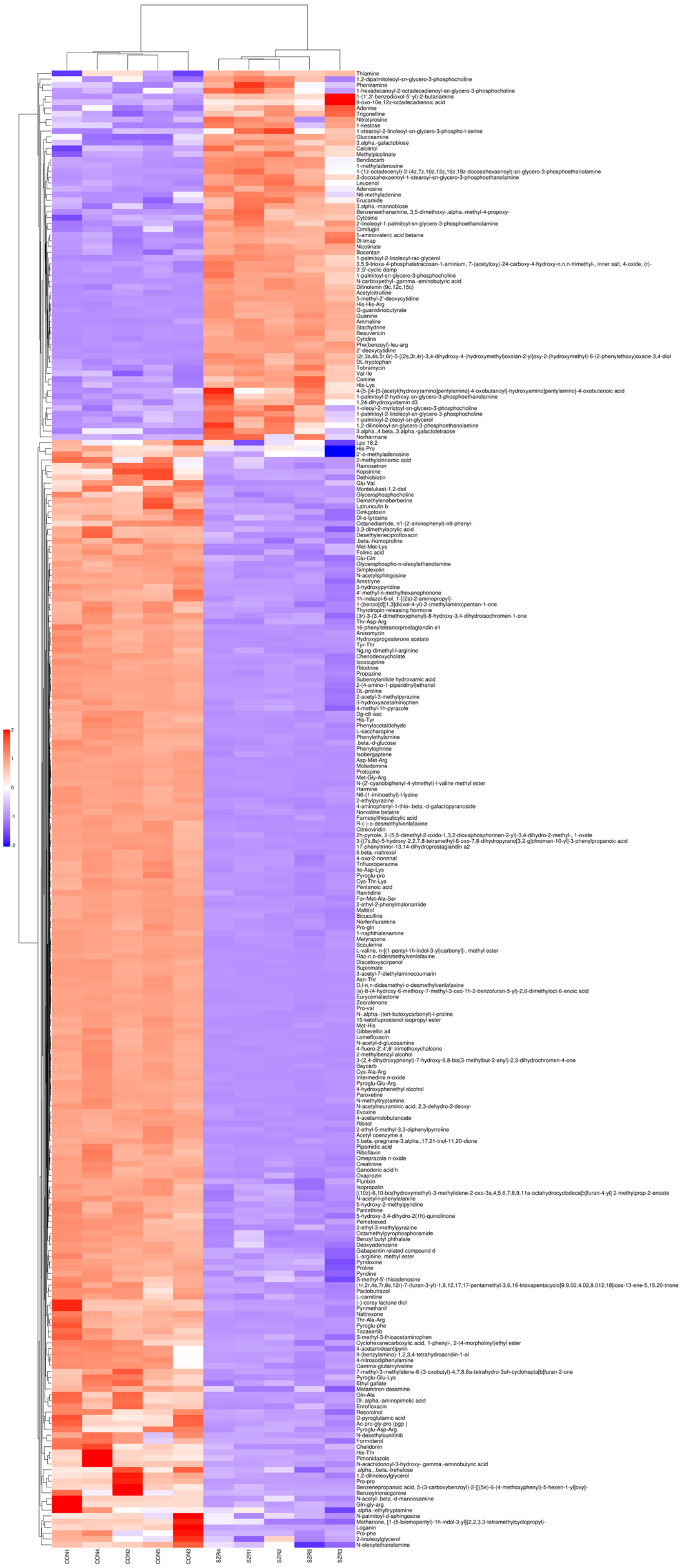

Figure S7: Heat map of hierarchical clustering of significantly different metabolites. Note: Each row in the figure represents a differential metabolite, and each column represents a set of samples. Red represents significant up-regulation, blue represents significant down-regulation, and color shades indicate the degree of up- and down-regulation; metabolites with close expression patterns are clustered under the same cluster on the left.

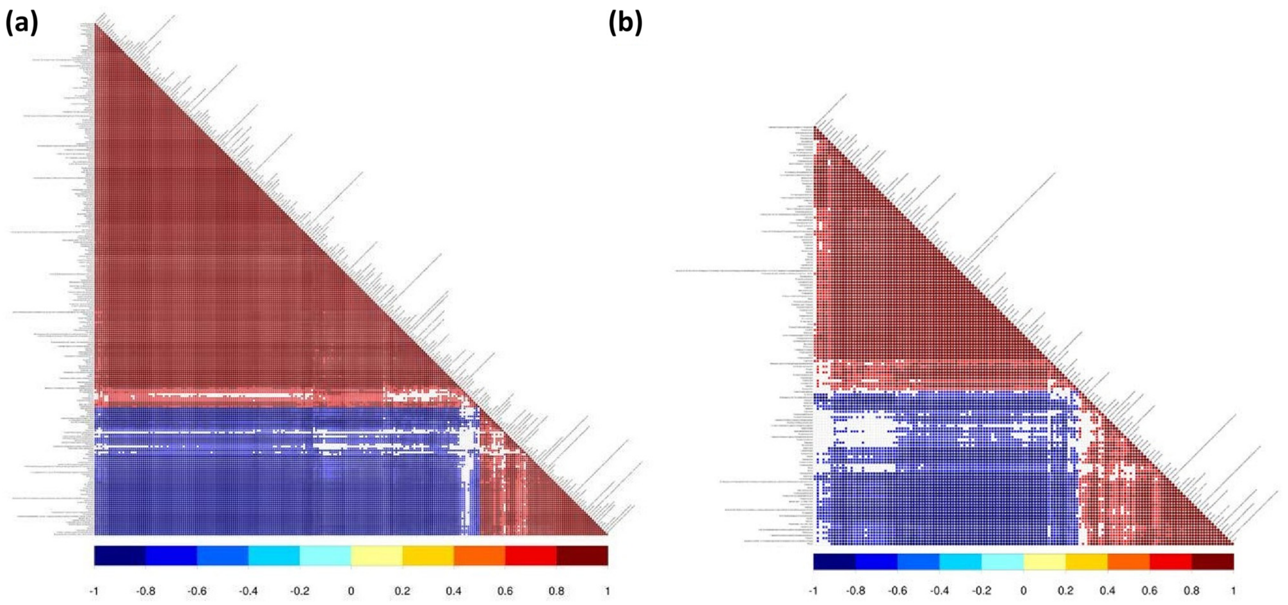

Figure S8: The results of the correlation analysis are visualized in the form of a correlation heat map. Note: Red indicates positive correlation, blue indicates negative correlation, and white indicates non-significant correlation. The color shade is related to the absolute magnitude of the correlation coefficient, i.e., the higher the degree of positive or negative correlation, the darker the color. The size of the dot is related to the significance of the correlation, the more significant, the smaller the *p*-value, the larger the dot.

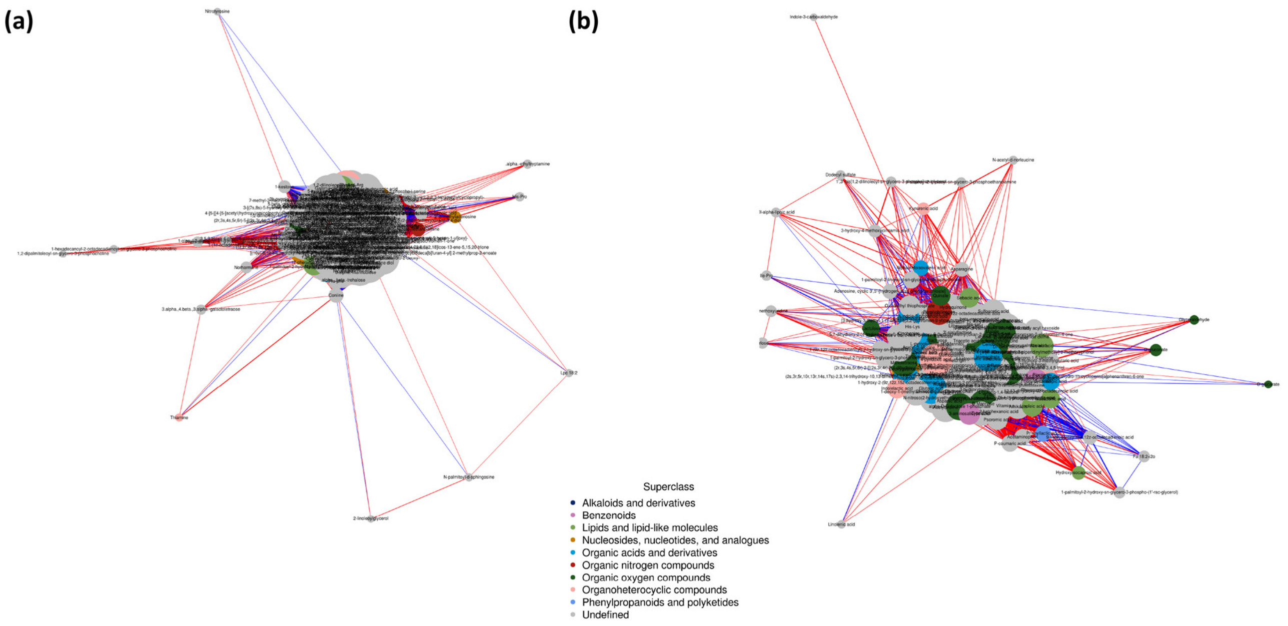

Figure S9: Network diagram between metabolites. (a) Positive ion mode network diagram. (b) Negative ion mode network diagram. Note: The dots in the graph represent significantly different metabolites, and the size of the dots correlates with the degree of connectivity; the larger the degree, the larger the dots. The color of the line represents the correlation, red indicates positive correlation, blue indicates negative correlation. The thickness of the line represents the absolute value of the correlation coefficient, the thicker the line, the larger the correlation.

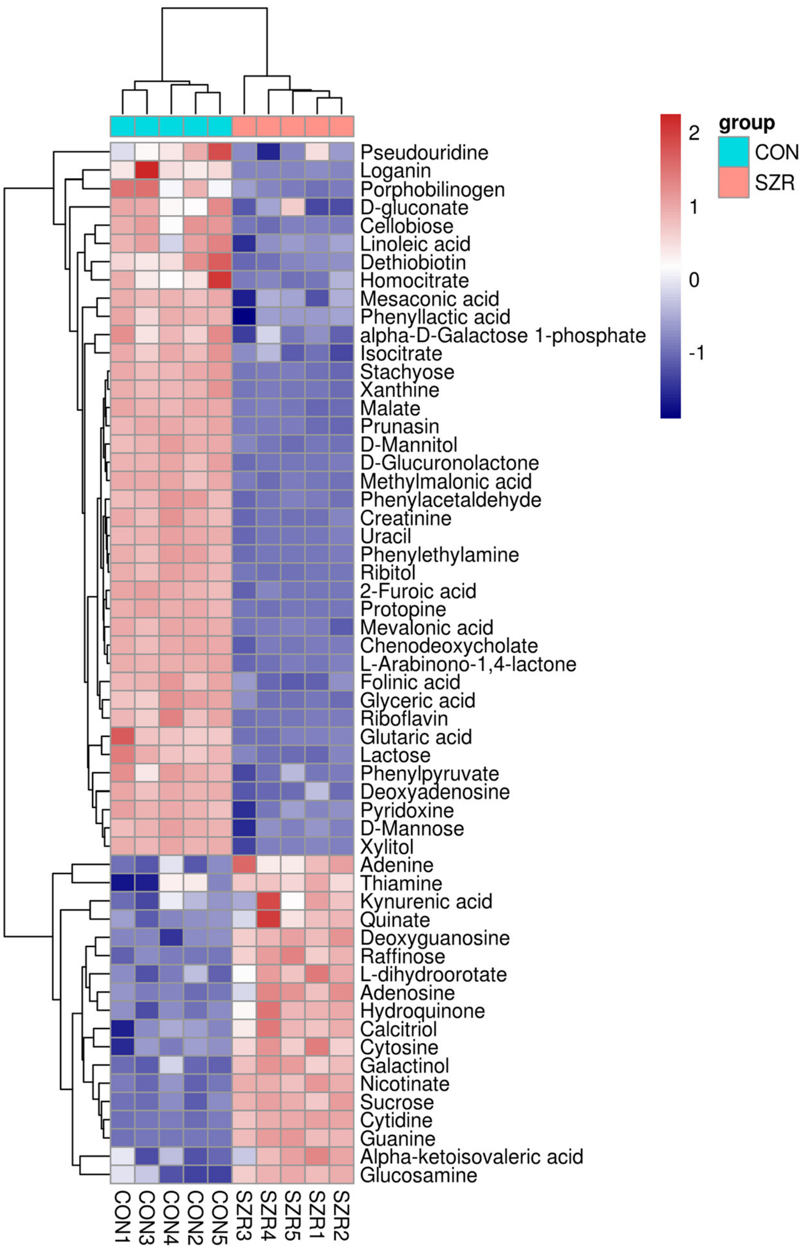

Figure S10: Heat map of differential metabolite clustering in the KEGG pathway.
